# Stochastic variational principles for the collisional Vlasov–Maxwell and Vlasov–Poisson equations

**DOI:** 10.1098/rspa.2021.0167

**Published:** 2021-08

**Authors:** Tomasz M. Tyranowski

**Affiliations:** ^1^ Max-Planck-Institut für Plasmaphysik, Boltzmannstraße 2, Garching 85748, Germany; ^2^ Technische Universität München, Zentrum Mathematik, Boltzmannstraße 3, Garching 85748, Germany

**Keywords:** stochastic variational principles, Vlasov–Maxwell equation, Vlasov–Poisson equation, collisions, Fokker–Planck equation, particle methods

## Abstract

In this work, we recast the collisional Vlasov–Maxwell and Vlasov–Poisson equations as systems of coupled stochastic and partial differential equations, and we derive stochastic variational principles which underlie such reformulations. We also propose a stochastic particle method for the collisional Vlasov–Maxwell equations and provide a variational characterization of it, which can be used as a basis for a further development of stochastic structure-preserving particle-in-cell integrators.

## 1. Introduction

The collisional Vlasov equation

1.1 $$\frac{\partial f}{\partial t}+\text{v}\cdot \nabla_{x}f+\frac{q}{m}(\text{E}+\text{v}\times \text{B})\cdot \nabla_{v}f=C[f],$$

describes the time evolution of the particle density function f=f(x,v,t) of plasma consisting of charged particles of charge *q* and mass *m* which undergo collisions described by the collision operator C[f], and are subject to the electric E=E(x,t) and magnetic B=B(x,t) fields. The vectors $\text{x}=(x^{1},x^{2},x^{3})$ and $\text{v}=(v^{1},v^{2},v^{3})$ denote positions and velocities, respectively. For simplicity, we restrict ourselves to one-piece plasmas. Usually, the particle density function is normalized, so that the total number of particles is $N_{\text{tot}}=\iint f(\text{x},\text{v},t){\text{d}}^{3}\text{v}{\text{d}}^{3}\text{x}$. However, in this work we would like to treat *f* as a probability density function, and therefore we will use the normalization $\iint f(\text{x},\text{v},t){\text{d}}^{3}\text{v}{\text{d}}^{3}\text{x}=1$ instead. A self-consistent model of plasma is obtained by coupling (1.1) with the Maxwell equations

1.2*a* $$\begin{array}{r} \nabla_{x}\cdot \text{E}=\rho , \end{array}$$

1.2*b* $$\begin{array}{r} \nabla_{x}\cdot \text{B}=0, \end{array}$$

1.2*c* $$\begin{array}{r} \nabla_{x}\times \text{E}=-\frac{\partial \text{B}}{\partial t} \end{array}$$

1.2*d* $$\begin{array}{r} \;\text{and}\;\nabla_{x}\times \text{B}=\frac{\partial \text{E}}{\partial t}+\text{J}, \end{array}$$

where

1.3 $$\rho (\text{x},t)=qN_{\text{tot}}\int_{R^{3}}f(\text{x},\text{v},t)\;{\text{d}}^{3}\text{v}\;\text{and}\;\text{J}(\text{x},t)=qN_{\text{tot}}\int_{R^{3}}\text{v}f(\text{x},\text{v},t)\;{\text{d}}^{3}\text{v},$$

denote the charge density and the electric current density, respectively, and the factor $N_{\text{tot}}$ is due to our normalization. The system (1.1)–(1.3) is usually referred to as the Vlasov–Maxwell equations. It will also be convenient to express the electric and magnetic fields in terms of the scalar φ(x,t) and vector A(x,t) potentials

1.4*a* $$\text{E}=-\nabla_{x}\varphi -\frac{\partial \text{A}}{\partial t}$$

and

1.4*b* $$\text{B}=\nabla_{x}\times \text{A},$$

as is typical in electrodynamics. The Vlasov–Poisson equations are an approximation of the Vlasov–Maxwell equations in the non-relativistic zero-magnetic field limit (see §6). The main goal of this work is to provide a variational characterization of the Vlasov–Maxwell and Vlasov–Poisson equations via a stochastic Lagrange–d’Alembert type of a principle.

Variational principles have proved extremely useful in the study of nonlinear evolution partial differential equations (PDEs). For instance, they often provide physical insights into the problem being considered; facilitate discovery of conserved quantities by relating them to symmetries via Noether’s theorem; allow one to determine approximate solutions to PDEs by minimizing the action functional over a class of test functions (e.g. [1]); and provide a way to construct a class of numerical methods called variational integrators [2,3]. A variational principle for the collisionless Vlasov–Maxwell equations was first proposed in [4]. It has been used to derive various particle discretizations of the Vlasov–Maxwell and Vlasov–Poisson equations [5–9], including structure-preserving variational particle-in-cell (PIC) methods [10–12]. It has also been applied to gyrokinetic theory (e.g. [13,14]). For other formulations and extensions, see also [15].

A structure-preserving description of collisional effects is far less developed. A metriplectic framework for the Vlasov–Maxwell-Landau equations has been presented in [16,17]. More recently, a stochastic variational principle has been proposed in [18] to describe collisional effects for the Vlasov equation with a fixed external electric field. To the best of our knowledge, to date no variational principle has been derived for the collisional Vlasov–Maxwell and Vlasov–Poisson equations. In this work, we extend the notion of the stochastic Lagrange–d’Alembert principle presented in [18] to plasmas evolving in self-consistent electromagnetic fields. The main idea of our approach is to interpret the Vlasov equation (1.1) as a Fokker–Planck equation and consider the associated stochastic differential equations.

The idea of using stochastic differential equations to model collisions has been pursued by a number of authors over the last few decades (e.g. [18–32], Y Fu, X Zhang, H Qin 2020, unpublished data).

There has been an ever-growing body of the literature dedicated to stochastic variational principles in recent years. Stochastic variational principles allow the introduction of noise into systems in such a way that the resulting probabilistic models retain all or some of the geometric properties of their deterministic counterparts. For this reason, stochastic variational principles have been considered in the context of Lagrangian and Hamiltonian mechanics [18,33–39], soliton dynamics [40,41], fluid dynamics [42–49] and kinetic plasma theory [18].

*Main content*. The main content of the remainder of this paper is, as follows.

In §2, we recast the collisional Vlasov–Maxwell equations as a system of coupled stochastic and partial differential equations.
In §3, we discuss the relationship between particle methods and stochastic modelling. We formulate a stochastic particle discretization for the collisional Vlasov–Maxwell equations and cast it in a form that allows the derivation of a variational principle.
In §4, we describe the variational structure underlying the stochastic particle discretization of the Vlasov–Maxwell system. The main result of this section is theorem 4.2, in which a stochastic Lagrange–d’Alembert principle for the particle discretization is proved.
In §5, we generalize the ideas from §4 to the original undiscretized equations. The main result of this section is theorem 5.1, in which a stochastic Lagrange–d’Alembert principle is proved for a class of the collisional Vlasov–Maxwell equations.
In §6, we prove a stochastic Lagrange–d’Alembert principle applicable to the Vlasov–Poisson equations. The main result of this section is theorem 6.1.
Section 7 contains the summary of our work.

## 2. The Vlasov–Maxwell–Fokker–Planck equations

### (a) Stochastic reformulation

Various collision models and various forms of the collision operator C[f] are considered in the plasma physics literature (e.g. [50,51]). A key step towards a stochastic variational principle is a probabilistic interpretation of the Vlasov equation (1.1). Therefore, in this work we will be interested only in those collision operators for which (1.1) takes the form of a linear or strongly nonlinear Fokker–Planck equation (e.g. [52–54]). Namely, we will assume that the collision operator can be expressed as

2.1 $$C[f]=\frac{1}{2}\sum_{i,j=1}^{3}\frac{\partial^{2}}{\partial v^{i}\partial v^{j}}[D_{ij}(\text{x},\text{v};f)f]-\sum_{i=1}^{3}\frac{\partial }{\partial v^{i}}[K_{i}(\text{x},\text{v};f)f],$$

for some symmetric positive semi-definite matrix $D_{ij}(\text{x},\text{v};f)$ and vector $K_{i}(\text{x},\text{v};f)$ functions, where the dependence of $D_{ij}$ and $K_{i}$ on *f* may in general be nonlinear, and may involve differential and integral forms of *f*. In that case (1.1) is an integro-differential equation, the so-called strongly nonlinear Fokker–Planck equation [52]. In case $D_{ij}$ and $K_{i}$ are independent of *f*, that is, $D_{ij}(\text{x},\text{v};f)=D_{ij}(\text{x},\text{v})$ and $K_{i}(\text{x},\text{v};f)=K_{i}(\text{x},\text{v})$, the Vlasov equation (1.1) reduces to the standard linear Fokker–Planck equation. We will further assume that $D_{ij}$ and $K_{i}$ can be expressed in the form

2.2 $$D_{ij}(\text{x},\text{v};f)=\sum_{\nu =1}^{M}g_{\nu }^{i}g_{\nu }^{j}\;\text{and}\;K_{i}(\text{x},\text{v};f)=G^{i}+\frac{1}{2}\sum_{\nu =1}^{M}\sum_{j=1}^{3}\frac{\partial g_{\nu }^{i}}{\partial v^{j}}g_{\nu }^{j},$$

for a vector function G(x,v;f), and a family of vector functions ${\text{g}}_{\nu }(\text{x},\text{v};f)$ with ν=1,…,M. Note that given a symmetric positive semi-definite matrix $D_{ij}$, a decomposition (2.2) can always be found, but it may not be unique. For instance, one may take M=3 and assume that $g_{\nu }^{i}=g_{i}^{\nu }$ for i,ν=1,2,3. Then the first equation in (2.2) implies that the family of functions $g_{\nu }^{i}$ can be determined by calculating the square root of the matrix $D_{ij}$, and the second equation in (2.2) can be used to calculate the function **G**. If (1.1) has the form of a Fokker–Planck equation, then the particle density function *f* can be interpreted as the probability density function for a stochastic process $(\text{X}(t),\text{V}(t))\in R^{3}\times R^{3}$. This stochastic process then satisfies the Stratonovich stochastic differential equation [52–55]

2.3*a* dX=V dt

and

2.3*b* $$\text{d}\text{V}=(\frac{q}{m}\text{E}(\text{X},t)+\frac{q}{m}\text{V}\times \text{B}(\text{X},t)+\text{G}(\text{X},\text{V};f))\;\text{d}t+\sum_{\nu =1}^{M}{\text{g}}_{\nu }(\text{X},\text{V};f)\circ \text{d}W^{\nu }(t),$$

where $W^{1}(t),\ldots ,W^{M}(t)$ denote the components of the standard *M*-dimensional Wiener process and ○ denotes Stratonovich integration. Note that the terms **G** and ${\text{g}}_{\nu }$ can be interpreted as external forces, and that in their absence the equations (2.3) reduce to the equations of motion of a charged particle in an electromagnetic field. We will therefore refer to **G** and ${\text{g}}_{\nu }$ as forcing terms. The electric and magnetic fields are coupled via the Maxwell equations (1.2). It should also be noted that unless (1.1) is linear, the right-hand side of (2.3) depends on *f*. In order to obtain a self-consistent system, one can express *f* in terms of the stochastic processes **X** and **V** as f(x,v,t)=E[δ(x−X(t))δ(v−V(t))], where E denotes the expected value, and δ is Dirac’s delta. This can be further plugged into (1.3). Together, we get

2.4*a* $$\begin{array}{rl} f(\text{x},\text{v},t) & =E[\delta (\text{x}-\text{X}(t))\delta (\text{v}-\text{V}(t))], \end{array}$$

2.4*b* $$\begin{array}{rl} \rho (\text{x},t) & =qN_{\text{tot}}E[\delta (\text{x}-\text{X}(t))] \end{array}$$

2.4*c* $$\begin{array}{rl} \;\text{and}\;\text{J}(\text{x},t) & =qN_{\text{tot}}E[\text{V}(t)\delta (\text{x}-\text{X}(t))]. \end{array}$$

Equations (1.2), (2.3) and (2.4) form a self-consistent system of stochastic and partial differential equations whose solutions are the stochastic processes X(t), V(t), and the functions E(x,t), B(x,t).

**Remark.**

Upon substituting (2.4*a*), the forcing terms **G** and ${\text{g}}_{\nu }$ become functionals of the processes **X** and **V**, that is, G(x,v;f)=G(x,v;X,V) and ${\text{g}}_{\nu }(\text{x},\text{v};f)={\text{g}}_{\nu }(\text{x},\text{v};\text{X},\text{V})$. However, for convenience and simplicity, throughout this work we will stick to the notation G(x,v;f) and ${\text{g}}_{\nu }(\text{x},\text{v};f)$, understanding that the probability density is given by (2.4*a*) (or by (3.2*a*) for particle discretizations; see §3).

### (b) Examples

Below we list a few examples of collision operators that fit the description presented in §2a.

#### (i) Lenard–Bernstein operator

The Lenard–Bernstein collision operator

2.5 $$C[f]=\nu_{c}(\mu \nabla_{v}\cdot (\text{v}f)+\frac{\gamma^{2}}{2}\Delta_{v}f),$$

where $\nu_{c}>0$, μ>0 and γ>0 are parameters, models small-angle collisions, and was originally used to study longitudinal plasma oscillations [50,51,56]. It can be easily verified that an example decomposition (2.2) for M=3 is given by the functions

2.6 $$\begin{array}{rl} & \text{G}(\text{x},\text{v})=-\nu_{c}\mu \text{v},\;{\text{g}}_{1}(\text{x},\text{v})=\left(\begin{array}{c} \sqrt{\nu_{c}}\gamma \\ 0\\ 0 \end{array}\right),\\ & {\text{g}}_{2}(\text{x},\text{v})=\left(\begin{array}{c} 0\\ \sqrt{\nu_{c}}\gamma \\ 0 \end{array}\right)\;\text{and}\;{\text{g}}_{3}(\text{x},\text{v})=\left(\begin{array}{c} 0\\ 0\\ \sqrt{\nu_{c}}\gamma \end{array}\right). \end{array}\}$$

Note that these functions do not explicitly depend on *f*, therefore in this case (1.1) is a linear Fokker–Planck equation.

#### (ii) Lorentz operator

The Lorentz collision operator models electron–ion interactions via pitch-angle scattering and is given by the formula

2.7 $$C[f]=\frac{\nu_{c}(|\text{v}|)}{2}\nabla_{v}\cdot (|\text{v}{|}^{2}I-\text{v}⊗\text{v})\nabla_{v}f,$$

where $\nu_{c}(|\text{v}|)$ is the collisional frequency as a function of the absolute value of velocity, I is the 3×3 identity matrix and ⊗ denotes tensor product. The primary effect of this type of scattering is a change of the direction of the electron’s velocity with negligible energy loss. More information about the Lorentz collision operator, including the exact form of the collision frequency, can be found in, e.g. [50,51,57,58]. It can be verified by a straightforward calculation that an example decomposition (2.2) for M=3 is given by the functions

2.8 $$\begin{array}{rl} & \text{G}(\text{x},\text{v})=0,\;{\text{g}}_{1}(\text{x},\text{v})=\sqrt{\nu_{c}(|\text{v}|)}\left(\begin{array}{c} 0\\ -v^{3}\\ v^{2} \end{array}\right),\\ & {\text{g}}_{2}(\text{x},\text{v})=\sqrt{\nu_{c}(|\text{v}|)}\left(\begin{array}{c} v^{3}\\ 0\\ -v^{1} \end{array}\right)\;\text{and}\;{\text{g}}_{3}(\text{x},\text{v})=\sqrt{\nu_{c}(|\text{v}|)}\left(\begin{array}{c} -v^{2}\\ v^{1}\\ 0 \end{array}\right). \end{array}\}$$

Note that these functions do not explicitly depend on *f*, therefore also in this case (1.1) is a linear Fokker–Planck equation.

#### (iii) Coulomb/Landau operator

The more general Coulomb collision operator has the form (2.1) with

2.9 $$\begin{array}{rl} & D_{ij}(\text{x},\text{v};f)=N_{\text{tot}}\Gamma \int_{R^{3}}\frac{|\text{v}-\text{u}{|}^{2}\delta_{ij}-(v^{i}-u^{i})(v^{j}-u^{j})}{|\text{v}-\text{u}{|}^{3}}f(\text{x},\text{u},t)\;{\text{d}}^{3}\text{u}\\ \;\text{and}\; & K_{i}(\text{x},\text{v};f)=-2N_{\text{tot}}\Gamma \int_{R^{3}}\frac{v^{i}-u^{i}}{|\text{v}-\text{u}{|}^{3}}f(\text{x},\text{u},t)\;{\text{d}}^{3}\text{u}, \end{array}\}$$

where $N_{\text{tot}}$ appears due to our normalization of *f*, $\delta_{ij}$ is Kronecker’s delta, and $\Gamma =(4\pi q^{4}/m^{2})\ln\Lambda$, with ln⁡Λ denoting the so-called Coulomb logarithm. The Coulomb operator describes collisions in which the fundamental two-body force obeys an inverse square law, and makes the assumption that small-angle collisions are more important than collisions resulting in large momentum changes [50,51,59]. A decomposition (2.2) can be found, for example, via the procedure outlined in §2a. However, the expressions for **G** and ${\text{g}}_{\nu }$ are complicated, therefore we are not stating them here explicitly. Note that $D_{ij}$ and $K_{i}$ explicitly depend on *f*. Therefore, for the Coulomb operator the Vlasov equation (1.1) is a strongly nonlinear Fokker–Planck equation. Note also that $D_{ij}$ and $K_{i}$ can be explicitly written as functionals of the stochastic processes **X** and **V** as

2.10 $$\begin{array}{rl} & D_{ij}(\text{x},\text{v};\text{X},\text{V})=N_{\text{tot}}\Gamma \cdot E[\frac{|\text{v}-\text{V}(t){|}^{2}\delta_{ij}-(v^{i}-V^{i}(t))(v^{j}-V^{j}(t))}{|\text{v}-\text{V}(t){|}^{3}}\delta (\text{x}-\text{X}(t))]\\ \;\text{and}\; & K_{i}(\text{x},\text{v};\text{X},\text{V})=-2N_{\text{tot}}\Gamma \cdot E[\frac{v^{i}-V^{i}(t)}{|\text{v}-\text{V}(t){|}^{3}}\delta (\text{x}-\text{X}(t))]. \end{array}\}$$

The collision operator (2.1) with $D_{ij}$ and $K_{i}$ as in (2.9) can also be expressed in an equivalent, although more symmetric form, known as the Landau form of the Coulomb operator, or simply the Landau collision operator (e.g. [50]).

## 3. Stochastic particle discretization of the Vlasov–Maxwell equations

Particle modelling is one of the most popular numerical techniques for solving the Vlasov equation (e.g. [60,61]). In this section, we discuss the connections between particle methods and stochastic modelling.

The standard particle method for the collisionless Vlasov equation (1.1) (with C[f]=0) consists of substituting the Ansatz $f(\text{x},\text{v},t)=\sum_{a=1}^{N}w_{a}\delta (\text{x}-{\text{X}}_{a}(t))\delta (\text{v}-{\text{V}}_{a}(t))$ for the particle density function, and deriving the corresponding ordinary differential equations satisfied by the ‘particle’ positions ${\text{X}}_{a}(t)$ and velocities ${\text{V}}_{a}(t)$, which turn out to be the characteristic equations. Note that we did a qualitatively similar thing in §2a, where we turned the original collisional Vlasov equation into the system of stochastic differential equations (2.3), which in the absence of the forcing terms **G** and ${\text{g}}_{\nu }$ have the same form as the characteristic equations, and in fact the ‘particles’ ${\text{X}}_{a}(t)$ and ${\text{V}}_{a}(t)$ can be interpreted as realizations of the stochastic processes X(t) and V(t) for different elementary events ω∈Ω.

When the right-hand side of (2.3) does not depend on *f*, then (2.3) can in principle be solved numerically with the help of any standard stochastic numerical method (e.g. [55]), and each realization of the stochastic processes can be simulated independently of others. When the right-hand side of (2.3) depends on *f*, then all realizations of the stochastic processes have to be solved for simultaneously, so that at each time step the probability density function *f* can be numerically approximated (e.g. [52]). Such an approach, however, does not quite lend itself to a geometric formulation. Therefore, in order to be able to introduce a variational principle in §4, let us consider 2N stochastic processes ${\text{X}}_{1},{\text{V}}_{1},\ldots ,{\text{X}}_{N},{\text{V}}_{N}$, with each pair $\left({\text{X}}_{a},{\text{V}}_{a}\right)$ satisfying the stochastic differential system

3.1*a* $$\text{d}{\text{X}}_{a}={\text{V}}_{a}\;\text{d}t$$

and

3.1*b* $$\begin{array}{rl} \text{d}{\text{V}}_{a} & =(\frac{q}{m}\text{E}({\text{X}}_{a},t)+\frac{q}{m}{\text{V}}_{a}\times \text{B}({\text{X}}_{a},t)+\text{G}({\text{X}}_{a},{\text{V}}_{a};f))\;\text{d}t\\ & \;+\sum_{\nu =1}^{M}{\text{g}}_{\nu }({\text{X}}_{a},{\text{V}}_{a};f)\circ \text{d}W_{a}^{\nu }(t), \end{array}$$

for a=1,…,N, where ${\text{W}}_{a}=(W_{a}^{1},\ldots ,W_{a}^{M})$ are *N* independent *M*-dimensional Wiener processes. Note that the systems (3.1) are decoupled from each other for different values of a, and each system is driven by an independent Wiener process ${\text{W}}_{a}$. Therefore, the pairs $\left({\text{X}}_{a},{\text{V}}_{a}\right)$ for a=1,…,N are independent identically distributed stochastic processes, each with the probability density function *f* that satisfies the original Fokker–Planck equation (1.1). In that sense (3.1) is equivalent to (2.3). The advantage is that instead of considering *N* realizations of the six-dimensional stochastic process (X,V) in (2.3), one can consider one realization of the 6N-dimensional process $\left({\text{X}}_{1},{\text{V}}_{1},\ldots ,{\text{X}}_{N},{\text{V}}_{N}\right)$ in (3.1). Such a reformulation will allow us to identify an underlying stochastic variational principle in §4. The last step leading to the stochastic particle discretization is approximating the probability density function *f* in (3.1). This can be done with the help of the law of large numbers, namely, one can approximate (2.4) for large *N* as

3.2*a* $$\begin{array}{rl} & f(\text{x},\text{v},t)\approx \frac{1}{N}\sum_{a=1}^{N}\delta (\text{x}-{\text{X}}_{a}(t))\delta (\text{v}-{\text{V}}_{a}(t)), \end{array}$$

3.2*b* $$\begin{array}{rl} & \rho (\text{x},t)\approx \frac{qN_{\text{tot}}}{N}\sum_{a=1}^{N}\delta (\text{x}-{\text{X}}_{a}(t)) \end{array}$$

3.2*c* $$\begin{array}{rl} \;\text{and}\; & \text{J}(\text{x},t)\approx \frac{qN_{\text{tot}}}{N}\sum_{a=1}^{N}{\text{V}}_{a}(t)\delta (\text{x}-{\text{X}}_{a}(t)). \end{array}$$

It is easy to see that (3.2*a*) coincides with the standard Ansatz used in particle modelling (with the weights $w_{a}=1/N$). Therefore, the system of stochastic differential equations (3.1) with the approximation (3.2), and with the electromagnetic field coupled via the Maxwell equations (1.2), can be considered as a stochastic particle discretization of the collisional Vlasov–Maxwell equations.

**Remark.**

Upon substituting (3.2*a*), the forcing terms **G** and ${\text{g}}_{\nu }$ become functionals of the processes ${\text{X}}_{1},\ldots ,{\text{X}}_{N}$ and ${\text{V}}_{1},\ldots ,{\text{V}}_{N}$. Similar to the discussion in §2a, for convenience and simplicity, throughout this work we will stick to the notation G(x,v;f) and ${\text{g}}_{\nu }(\text{x},\text{v};f)$, understanding that the probability density is given by (3.2*a*) for particle discretizations.

## 4. Variational principle for the particle discretization

In this section, we propose an action functional which can be understood as a stochastic version of the Low action functional [4], and we prove a variational principle underlying the particle discretization introduced in §3, akin to the stochastic Lagrange–d’Alembert principle first introduced in [18].

### (a) Function spaces

Before we introduce the action functional, we need to identify suitable function spaces on which it will be defined. For simplicity, let our spatial domain be the whole three-dimensional space $R^{3}$, and let us consider the time interval [0,T] for some T>0. Let (Ω,F,P) be the probability space with the filtration ${\left\{F_{t}\right\}}_{t\geq 0}$, and let ${\text{W}}_{a}=(W_{a}^{1},\ldots ,W_{a}^{M})$ for a=1,…,N denote *N* independent *M*-dimensional Wiener processes on that probability space (such that $W_{a}^{\nu }(t)$ is $F_{t}$-measurable for all t≥0). The stochastic processes ${\text{X}}_{a}(t)$ and ${\text{V}}_{a}(t)$ satisfy (3.1), so they are in particular $F_{t}$-adapted semimartingales, and have almost surely continuous paths [62]. We also notice that there is no diffusion term in (3.1), therefore we even have that the processes ${\text{X}}_{a}(t)$ are almost surely of class $C^{1}$. We introduce the notation

4.1 $$\begin{array}{rl} C_{\Omega ,T}^{k} & =\{\text{X}\in L^{2}(\Omega \times [0,T],R^{3})|\text{X}\;\text{is a}\;F_{t}\text{-adapted semimartingale},\\ & \;\text{almost surely of class}\;C^{k}\}. \end{array}$$

Note that this set is a vector space [62]. The potentials φ and **A** satisfy the Maxwell equations (1.2) and (1.4), therefore we require them to be of class $C^{2}$. However, since our spatial domain is unbounded, we further need to assume that the vector fields **E** and **B** are square integrable. We introduce the notation

4.2 $$\begin{array}{rl} X(R^{n}) & =\{\text{A}\in C^{2}(R^{3}\times [0,T],R^{n})\cap L^{\infty }(R^{3}\times [0,T],R^{n})|\\ & \;\forall i,j:\frac{\partial A^{i}}{\partial x^{j}},\frac{\partial A^{i}}{\partial t}\in L^{2}(R^{3}\times [0,T])\}\\ \;\text{and}\;X_{0}(R^{n}) & =C_{0}^{2}(R^{3}\times [0,T],R^{n}), \end{array}\}$$

where $X_{0}(R^{n})$ is simply the space of compactly supported elements of $X(R^{n})$.

### (b) Action functional

Let us consider the action functional

4.3 $$S:\Omega \times (C_{\Omega ,T}^{1}{)}^{N}\times (C_{\Omega ,T}^{0}{)}^{N}\times (C_{\Omega ,T}^{0}{)}^{N}\times X(R)\times X(R^{3})⟶R,$$

defined by the formula

4.4 $$\begin{array}{rl} & S[{\text{X}}_{1},\ldots ,{\text{X}}_{N},{\text{V}}_{1},\ldots ,{\text{V}}_{N},{\text{P}}_{1},\ldots ,{\text{P}}_{N},\varphi ,\text{A}]\\ & \;=\frac{N_{\text{tot}}}{N}\sum_{a=1}^{N}[\int_{0}^{T}(\frac{m}{2}|{\text{V}}_{a}{|}^{2}-q\varphi ({\text{X}}_{a},t)+q{\text{V}}_{a}\cdot \text{A}({\text{X}}_{a},t)+{\text{P}}_{a}\cdot ({\dot{\text{X}}}_{a}-{\text{V}}_{a}))\;\text{d}t]\\ & \;\;+\int_{0}^{T}\int_{R^{3}}\frac{1}{2}(|\text{E}{|}^{2}-|\text{B}{|}^{2})\;{\text{d}}^{3}\text{x}\text{d}t, \end{array}$$

where ${\dot{\text{X}}}_{a}$ denotes the time derivative of ${\text{X}}_{a}$, and the electric and magnetic fields **E** and **B** are expressed in terms of the partial derivatives of the potentials φ and **A** as in (1.4). Following the standard convention in stochastic analysis, we will omit writing elementary events ω∈Ω as arguments of stochastic processes unless otherwise needed, i.e. ${\text{X}}_{a}(t)\equiv {\text{X}}_{a}(\omega ,t)$. The action functional (4.4) resembles the Low action functional introduced in [4]. In fact, it can be viewed as a particle discretization of the Low action functional, written in terms of stochastic processes [5,6,8,10–12]. The term ${\text{P}}_{a}\cdot ({\dot{\text{X}}}_{a}-{\text{V}}_{a})$ is the so-called Hamilton–Pontryagin kinematic constraint (e.g. [63,64]) that enforces that ${\dot{\text{X}}}_{a}={\text{V}}_{a}$ using the Lagrange multiplier ${\text{P}}_{a}$, which turns out to be the conjugate momentum. In principle, this constraint is not necessary in our context—we could omit it and replace ${\text{V}}_{a}$ with ${\dot{\text{X}}}_{a}$ in (4.4). We will, however, keep it in order to make a clear connection with the theory developed in [35]. It also makes the notation in the proof of the stochastic Lagrange–d’Alembert principle in §4c more convenient and elegant. Note that the action functional *S* is itself a random variable, as ω∈Ω is one of its arguments. The variations of *S* with respect to its arguments are given by (see appendix A for the details of the derivations)

4.5*a* $$\begin{array}{rl} \delta_{{\text{X}}_{a}}S & =\frac{N_{\text{tot}}}{N}({\text{P}}_{a}(T)\cdot \delta {\text{X}}_{a}(T)-{\text{P}}_{a}(0)\cdot \delta {\text{X}}_{a}(0))\\ & \;+\frac{N_{\text{tot}}}{N}[-\int_{0}^{T}\delta {\text{X}}_{a}\circ \text{d}{\text{P}}_{a}+\int_{0}^{T}(-q\nabla_{x}\varphi ({\text{X}}_{a},t)\cdot \delta {\text{X}}_{a}\\ & \;+q\sum_{i,j=1}^{3}V^{j}\frac{\partial A^{j}}{\partial x^{i}}({\text{X}}_{a},t)\delta X_{a}^{i})\;\text{d}t], \end{array}$$

4.5*b* $$\begin{array}{rl} \delta_{{\text{V}}_{a}}S & =\frac{N_{\text{tot}}}{N}\int_{0}^{T}(m{\text{V}}_{a}+q\text{A}({\text{X}}_{a},t)-{\text{P}}_{a})\cdot \delta {\text{V}}_{a}\;\text{d}t, \end{array}$$

4.5*c* $$\begin{array}{rl} \delta_{{\text{P}}_{a}}S & =\frac{N_{\text{tot}}}{N}\int_{0}^{T}({\dot{\text{X}}}_{a}-{\text{V}}_{a})\cdot \delta {\text{P}}_{a}\;\text{d}t, \end{array}$$

4.5*d* $$\begin{array}{rl} \delta_{\text{A}}S & =\int_{0}^{T}\int_{R^{3}}(\text{J}+\frac{\partial \text{E}}{\partial t}-\nabla_{x}\times \text{B})\cdot \delta \text{A}\;{\text{d}}^{3}\text{x}\text{d}t\\ & \;-\int_{R^{3}}(\text{E}(\text{x},T)\cdot \delta \text{A}(\text{x},T)-\text{E}(\text{x},0)\cdot \delta \text{A}(\text{x},0))\;{\text{d}}^{3}\text{x} \end{array}$$

4.5*e* $$\begin{array}{r} \;\text{and}\;\delta_{\varphi }S=\int_{0}^{T}\int_{R^{3}}(\nabla_{x}\cdot \text{E}-\rho )\cdot \delta \varphi \;{\text{d}}^{3}\text{x}\text{d}t, \end{array}$$

where *ρ* and **J** are defined in (3.2*b*) and (3.2*c*), respectively. The total variation of *S* with respect to the variations of all arguments equals

4.6 $$\delta S=\sum_{a=1}^{N}(\delta_{{\text{X}}_{a}}S+\delta_{{\text{V}}_{a}}S+\delta_{{\text{P}}_{a}}S)+\delta_{\varphi }S+\delta_{\text{A}}S.$$

### (c) The stochastic Lagrange–d’Alembert principle

While the standard rules of the calculus of variations apply to the variations (4.5*d*,*e*), the variations (4.5*a*–*c*) involve stochastic processes and stochastic integrals. Therefore, before we can formulate a stochastic variational principle, we need the following lemma, whose proof is given in appendix B.

**Lemma 4.1.**

*Let* $X\in C_{\Omega ,T}^{1}$ *and* $V,P\in C_{\Omega ,T}^{0}$, *and let* $R,r_{\nu }:R^{3}\times R^{3}⟶R^{3}$ *be of class* $C^{1}$ *for* ν=1,…,M. *Then*

4.7 $$\begin{array}{rl} & \forall Z\in C_{\Omega ,T}^{1}:\int_{0}^{T}(Z(t)\circ \text{d}P-R(X,V)\cdot Z(t)\;\text{d}t\\ & \;-\sum_{\nu =1}^{M}r_{\nu }(X,V)\cdot Z(t)\circ \text{d}W^{\nu }(t))=0\;\text{a.s.}, \end{array}$$

*if and only if*

4.8 $$\begin{array}{rl} & \forall t\in [0,T]:\int_{0}^{t}(\text{d}P(\tau )-R(X(\tau ),V(\tau ))\;\text{d}\tau \\ & \;-\sum_{\nu =1}^{M}r_{\nu }(X(\tau ),V(\tau ))\circ \text{d}W^{\nu }(\tau ))=0\;\text{a.s.}, \end{array}$$

*where ‘a.s.’ means almost surely*.

**Remark.**

Equation (4.8) means that P(t), X(t) and V(t) satisfy a stochastic differential equation, which can be written in the differential form as

4.9 $$\text{d}\text{P}(t)=\text{R}(\text{X}(t),\text{V}(t))\;\text{d}t+\sum_{\nu =1}^{M}{\text{r}}_{\nu }(\text{X}(t),\text{V}(t))\circ \text{d}W^{\nu }(t).$$

We are now in a position to formulate and prove a stochastic variational principle that generalizes the deterministic Lagrange–d’Alembert principle for forced Lagrangian and Hamiltonian systems, akin to the stochastic variational principle introduced in [18].

**Theorem 4.2 (Stochastic Lagrange–d’Alembert principle for particles).**

*Let* $X_{a}\in C_{\Omega ,T}^{1}$ *and* $V_{a},P_{a}\in C_{\Omega ,T}^{0}$ *for* a=1,…,N *be stochastic processes, and let* $\text{A}\in X(R^{3})$, φ∈X(R) *be functions. Assume that* G(⋅,⋅;f) *and* ${\text{g}}_{\nu }(\cdot ,\cdot ;f)$ *for* ν=1,…,M *are* $C^{1}$ *functions of their arguments, where f is given by* (3.2*a*). *Then* $X_{a}$, $V_{a}$, $P_{a}$, A *and* φ *satisfy the system of stochastic differential equations*

4.10*a* $$\begin{array}{rl} & {\dot{X}}_{a}=V_{a}, \end{array}$$

4.10*b* $$\begin{array}{rl} & P_{a}=mV_{a}+qA(X_{a},t) \end{array}$$

4.10*c* $$\begin{array}{rl} \;\text{and}\; & \text{d}P_{a}^{i}=(-q\frac{\partial \varphi }{\partial x^{i}}(X_{a},t)+q\sum_{j=1}^{3}V_{a}^{j}\frac{\partial A^{j}}{\partial x^{i}}(X_{a},t)+m\;G^{i}(X_{a},V_{a};f))\;\text{d}t\\ & \;\;+m\sum_{\nu =1}^{M}g_{\nu }^{i}(X_{a},V_{a};f)\circ \text{d}W_{a}^{\nu }(t), \end{array}$$

*for* i=1,2,3 *and* a=1,…,N, *together with the Maxwell equations* (1.2), (1.4) *and* (3.2) *on the time interval* [0,T], *if and only if they satisfy the following variational principle*

4.11 $$\delta S+\frac{mN_{\text{tot}}}{N}\sum_{a=1}^{N}[\int_{0}^{T}G(X_{a},V_{a};f)\cdot \delta X_{a}\;\text{d}t+\sum_{\nu =1}^{M}\int_{0}^{T}g_{\nu }(X_{a},V_{a};f)\cdot \delta X_{a}\circ \text{d}W_{a}^{\nu }(t)]=0$$

*for arbitrary variations* $\delta X_{a}\in C_{\Omega ,T}^{1}$, $\delta V_{a},\delta P_{a}\in C_{\Omega ,T}^{0}$, $\delta A\in X_{0}(R^{3})$, *and* $\delta \varphi \in X_{0}(R)$, *with* $\delta X_{a}(0)=\delta X_{a}(T)=0$ *almost surely, and* δA(x,0)=δA(x,T)=0 *for all* $x\in R^{3}$, *where the action functional S is given by* (4.4).

**Proof.**

Let us first consider the variations with respect to **A** in (4.11). Given the boundary conditions for δA, from the standard calculus of variations we have that $\delta_{\text{A}}S=0$ (see equation (4.5*d*)) for all δA if and only if (1.2*d*) is satisfied. Similarly, $\delta_{\varphi }S=0$ (see equation (4.5*e*)) holds for all δφ if and only if (1.2*a*) holds. Further, for variations with respect to ${\text{V}}_{a}$ we have that $\delta_{{\text{V}}_{a}}S=0$ (see equation (4.5*b*)) for all $\delta {\text{V}}_{a}$ if and only if (4.10*b*) is satisfied almost surely, which follows from the standard theorem of the calculus of variations, since the integral in (4.5*b*) is a standard Lebesgue integral, and the integrands are almost surely continuous. Similarly, $\delta_{{\text{P}}_{a}}S=0$ (see equation (4.5*c*)) for all $\delta {\text{P}}_{a}$ if and only if (4.10*a*) is satisfied almost surely. Finally, for variations with respect to ${\text{X}}_{a}$, equations (4.5*a*) and (4.11) give

4.12 $$\begin{array}{rl} & \int_{0}^{T}(-\delta {\text{X}}_{a}\circ \text{d}{\text{P}}_{a}+(-q\nabla_{x}\varphi ({\text{X}}_{a},t)\cdot \delta {\text{X}}_{a}\\ & \;+q\sum_{i,j=1}^{3}V^{j}\frac{\partial A^{j}}{\partial x^{i}}({\text{X}}_{a},t)\delta X_{a}^{i}+m\text{G}({\text{X}}_{a},{\text{V}}_{a};f)\cdot \delta {\text{X}}_{a})\;\text{d}t\\ & \;+m\sum_{\nu =1}^{M}{\text{g}}_{\nu }({\text{X}}_{a},{\text{V}}_{a};f)\cdot \delta {\text{X}}_{a}\circ \text{d}W_{a}^{\nu }(t))=0, \end{array}$$

which, by lemma 4.1, holds for all $\delta {\text{X}}_{a}$ if and only if (4.10*c*) is satisfied.

**Remark.**

Equation (4.10) is expressed in terms of the Lagrange multipliers ${\text{P}}_{a}$, which, as can be seen in (4.10*b*), turn out to be the conjugate momenta. The conjugate momenta can be eliminated, and equation (4.10) can be recast as equation (3.1*b*), which is shown in the following theorem.

**Theorem 4.3.**

*Equations* (3.1) *and* (4.10) *are equivalent*.

**Proof.**

By calculating the stochastic differential on both sides of (4.10*b*) and substituting (4.10*a*), we obtain

4.13 $$\text{d}P_{a}^{i}=m\;\text{d}V_{a}^{i}+q\sum_{j=1}^{3}V_{a}^{j}\frac{\partial A^{i}}{\partial x^{j}}({\text{X}}_{a},t)\;\text{d}t+q\frac{\partial A^{i}}{\partial t}({\text{X}}_{a},t)\;\text{d}t,$$

for each i=1,2,3 and a=1,…,N. Comparing this with (4.10*c*), and using (1.4), one eliminates the conjugate momenta and obtains equation (3.1*d*).

**Remark.**

Theorems 4.2 and 4.3 provide a variational formulation of the stochastic particle method from §3. One can further perform a variational discretization of the electromagnetic fields **A** and φ, for instance along the lines of [10,65] or [66], thus obtaining a stochastic PIC discretization of the collisional Vlasov–Maxwell equations. The resulting structure-preserving numerical methods will be investigated in a follow-up work.

## 5. Variational principle for the Vlasov–Maxwell equations

The form of the action functional (4.4) and of the Lagrange–d’Alembert principle (4.11) suggests that it should be possible to formulate a similar variational principle for the stochastic reformulation of the Vlasov–Maxwell system discussed in §2a. In this section, we provide such a variational principle for a class of collision operators.

### (a) Action functional

Let us consider the action functional defined by the formula

5.1 $$\begin{array}{rl} \bar{S}[\text{X},\text{V},\text{P},\varphi ,\text{A}] & =N_{\text{tot}}\cdot E[\int_{0}^{T}(\frac{m}{2}|\text{V}{|}^{2}-q\varphi (\text{X},t)+q\text{V}\cdot \text{A}(\text{X},t)+\text{P}\cdot (\dot{\text{X}}-\text{V}))\text{d}t]\\ & \;+\int_{0}^{T}\int_{R^{3}}\frac{1}{2}(|\text{E}{|}^{2}-|\text{B}{|}^{2})\;{\text{d}}^{3}\text{x}\text{d}t, \end{array}$$

where $\dot{\text{X}}$ denotes the time derivative of **X**, the electric and magnetic fields **E** and **B** are expressed in terms of the partial derivatives of the potentials φ and **A** as in (1.4), and $E[Y]\equiv \int_{\Omega }Y\;\text{d}P$ denotes the expected value of the random variable Y. Note that unlike *S* in (4.4), the action functional $\bar{S}$ is not a random variable, as the dependence on ω∈Ω is integrated out with respect to the probability measure by calculating the expected value. In fact, *S* could be regarded as a Monte Carlo approximation of $\bar{S}$ when the processes ${\text{X}}_{1},\ldots ,{\text{X}}_{N}$ are independent and identically distributed as **X**, and similarly for **V** and **P**. An important issue to consider is the domain of this action functional. In a similar manner to (4.3), one may want to take as the domain the set

5.2 $$C_{\Omega ,T}^{1}\times C_{\Omega ,T}^{0}\times C_{\Omega ,T}^{0}\times X(R)\times X(R^{3}),$$

on which the formula (5.1) is well defined. This domain, however, turns out to be too big, in the sense that, as will be discussed below, due to the presence of the expected value the variations of $\bar{S}$ do not uniquely determine the set of stochastic evolution equations (2.3). It is therefore necessary to restrict (5.2) to a smaller subspace or submanifold which is compatible with the considered collision operator. Below we will demonstrate how this can be done for a class of collision operators (2.1) for which $D_{ij}(\text{x},\text{v};f)=\text{const}$, that is, we have

5.3 $${\text{g}}_{\nu }(\text{x},\text{v};f)=\chi \;\chi_{\nu }=\text{const}.$$

This class encompasses, for instance, the Lenard–Bernstein operator (2.6), or the more general nonlinear energy and momentum preserving Dougherty collision operator and its modifications [67–74]. For a given collision operator of the form (5.3), we define a compatible subset of $C_{\Omega ,T}^{0}$, namely,

5.4 $$C_{\text{col}}=\{\text{P}\in C_{\Omega ,T}^{0}|\exists \text{Z}\in C_{\Omega ,T}^{0}:\text{d}\text{P}=\text{Z}\;\text{d}t+m\sum_{\nu =1}^{M}\chi \;\chi_{\nu }\;\text{d}W^{\nu }(t)\}.$$

Note that for any ${\text{P}}_{1},{\text{P}}_{2}\in C_{\text{col}}$ we have that $d({\text{P}}_{1}-{\text{P}}_{2})=({\text{Z}}_{1}-{\text{Z}}_{2})\;\text{d}t$, that is, ${\text{P}}_{1}-{\text{P}}_{2}\in C_{\Omega ,T}^{1}$. Therefore, the pair $\left(C_{\text{col}},C_{\Omega ,T}^{1}\right)$ is an affine subspace of $C_{\Omega ,T}^{0}$. The action functional $\bar{S}$ can now be defined as

5.5 $$\bar{S}:C_{\Omega ,T}^{1}\times C_{\Omega ,T}^{0}\times C_{\text{col}}\times X(R)\times X(R^{3})⟶R.$$

Similar to the calculations in §4b, the variations of $\bar{S}$ with respect to **V** and **P** are given by, respectively,

5.6 $$\delta_{\text{V}}\bar{S}=N_{\text{tot}}\cdot E[\int_{0}^{T}(m\text{V}+q\text{A}(\text{X},t)-\text{P})\cdot \delta \text{V}\;\text{d}t]$$

and

5.7 $$\delta_{\text{P}}\bar{\text{S}}=N_{\text{tot}}\cdot E[\int_{0}^{T}(\dot{\text{X}}-\text{V})\cdot \delta \text{P}\;\text{d}t],$$

except that here $\delta \text{P}\in C_{\Omega ,T}^{1}$, so that $\text{P}+\epsilon \delta \text{P}\in C_{\text{col}}$. For the variation of $\bar{S}$ with respect to **X** we have

5.8 $$\begin{array}{rl} \delta_{\text{X}}\bar{S} & =N_{\text{tot}}\cdot E(\text{P}(T)\cdot \delta \text{X}(T)-\text{P}(0)\cdot \delta \text{X}(0))+N_{\text{tot}}\cdot E[-\int_{0}^{T}\delta \text{X}\circ \text{d}\text{P}\\ & \;+\int_{0}^{T}(-q\nabla_{x}\varphi (\text{X},t)\cdot \delta \text{X}+q\sum_{i,j=1}^{3}V^{j}\frac{\partial A^{j}}{\partial x^{i}}(\text{X},t)\delta X^{i})\;\text{d}t]. \end{array}$$

Since $\text{P}\in C_{\text{col}}$, we have that $\text{d}\text{P}=\text{Z}\;\text{d}t+m\sum_{\nu =1}^{M}\chi \;\chi_{\nu }\;\text{d}W^{\nu }(t)$. Furthermore, the variations δX are almost surely of class $C^{1}$, and therefore have sample paths of almost surely finite variation. Consequently, the quadratic covariation ${\left[\chi \;\chi_{\nu }\cdot \delta \text{X},W^{\nu }\right]}_{0}^{T}=0$ almost surely [62]. Since the expected value of the Itô integral with respect to the Wiener process is zero, we altogether have that

5.9 $$E[\int_{0}^{T}\chi \;\chi_{\nu }\cdot \delta \text{X}\circ \text{d}W^{\nu }(t)]=0,\;\text{for all }\nu =1,\ldots ,M.$$

By plugging this in (5.8), we finally obtain

5.10 $$\begin{array}{rl} \delta_{\text{X}}\bar{S} & =N_{\text{tot}}\cdot E(\text{P}(T)\cdot \delta \text{X}(T)-\text{P}(0)\cdot \delta \text{X}(0))\\ & \;+N_{\text{tot}}\cdot E[\int_{0}^{T}(-\text{Z}\cdot \delta \text{X}-q\nabla_{x}\varphi (\text{X},t)\cdot \delta \text{X}+q\sum_{i,j=1}^{3}V^{j}\frac{\partial A^{j}}{\partial x^{i}}(\text{X},t)\delta X^{i})\;\text{d}t]. \end{array}$$

The variations with respect to **A** and φ are the same as in (4.5*d*,*e*), respectively, only with the charge and electric current densities given by (2.4) rather than (3.2). The total variation of $\bar{S}$ with respect to the variations of all arguments is given by

5.11 $$\delta \bar{S}=\delta_{\text{X}}\bar{S}+\delta_{\text{V}}\bar{S}+\delta_{\text{P}}\bar{S}+\delta_{\varphi }\bar{S}+\delta_{\text{A}}\bar{S}.$$

### (b) The stochastic Lagrange–d’Alembert principle

In the following theorems, we establish a variational principle for the system of equations (1.2), (2.3) and (2.4) for a class of collision operators with ${\text{g}}_{\nu }(\text{x},\text{v};f)=\chi \;\chi_{\nu }=\text{const}$ for all ν=1,…,M.

**Theorem 5.1 (Stochastic Lagrange–d’Alembert principle for the VM equations).**

*Let* $X\in C_{\Omega ,T}^{1}$, $V\in C_{\Omega ,T}^{0}$, $P\in C_{\text{col}}$ *be stochastic processes, and let* $A\in X(R^{3})$, φ∈X(R) *be functions. Assume that* G(⋅,⋅;f) *is a* $C^{1}$ *function of its arguments, where f is given by* (2.4*a*). *Then* X, V, P, A *and* φ *satisfy the system of stochastic differential equations*

5.12*a* $$\begin{array}{rl} \dot{X} & =V, \end{array}$$

5.12*b* $$\begin{array}{rl} P & =mV+qA(X,t) \end{array}$$

5.12*c* $$\begin{array}{rl} \;\text{and}\;\text{d}P^{i} & =(-q\frac{\partial \varphi }{\partial x^{i}}(X,t)+q\sum_{j=1}^{3}V^{j}\frac{\partial A^{j}}{\partial x^{i}}(X,t)+mG^{i}(X,V;f))\;\text{d}t\\ & \;+m\sum_{\nu =1}^{M}\chi_{\nu }^{i}\;\text{d}W^{\nu }(t), \end{array}$$

*for* i=1,2,3, *together with the Maxwell equations* (1.2), (1.4) *and* (2.4) *on the time interval* [0,T], *if and only if they satisfy the following variational principle*

5.13 $$\delta \bar{S}+mN_{\text{tot}}\cdot E[\int_{0}^{T}G(X,V;f)\cdot \delta X\;\text{d}t]=0$$

*for arbitrary variations* $\delta X,\delta P\in C_{\Omega ,T}^{1}$, $\delta V\in C_{\Omega ,T}^{0}$, $\delta A\in X_{0}(R^{3})$, *and* $\delta \varphi \in X_{0}(R)$, *with* δX(0)=δX(T)=0 *almost surely, and* δA(x,0)=δA(x,T)=0 *for all* $x\in R^{3}$, *where the action functional* $\bar{S}$ *is given by* (5.1) *and* (5.5).

**Proof.**

Similar to the proof of theorem 4.2, the equations $\delta_{\varphi }\bar{S}=0$ and $\delta_{\text{A}}\bar{S}=0$ are equivalent to (1.2*a*) and (1.2*d*), respectively. Note that $C_{\Omega ,T}^{0}$ is a subspace of $L^{2}(\Omega \times [0,T],R^{3})$, and $\left\langle {\text{Y}}_{1},{\text{Y}}_{2}\rangle =E[\int_{0}^{T}{\text{Y}}_{1}\cdot {\text{Y}}_{2}\;\text{d}t\right]$ is an inner product on that space. Therefore, by substituting equations (5.6), (5.7) and (5.10) in equation (5.13), and using the fact that the variations are arbitrary, we establish equivalence with equations (5.12*a*,b), as well as with the equation

5.14 $$Z^{i}=-q\frac{\partial \varphi }{\partial x^{i}}(\text{X},t)+q\sum_{j=1}^{3}V^{j}\frac{\partial A^{j}}{\partial x^{i}}(\text{X},t)+mG^{i}(\text{X},\text{V};f),$$

for i=1,2,3, which in turn is equivalent to equation (5.12*c*), given the assumption $\text{P}\in C_{\text{col}}$.

**Theorem 5.2.**

*Equation* (2.3) *with* $g_{\nu }(x,v;f)=\chi \;\chi_{\nu }=\mathrm{const}$ *for* ν=1,…,M *and equation* (5.12) *are equivalent*.

**Proof.**

Similar to the proof of theorem 4.3, by calculating the stochastic differential on both sides of equation (5.12*b*) and comparing with equation (5.12*c*), one eliminates **P** and obtains equation (2.3*b*).

**Remark.**

Note that the forcing terms ${\text{g}}_{\nu }$ do not explicitly appear in the variational equation (5.13). By comparing theorem 4.2 and theorem 5.1, one could intuitively expect that the relevant variational principle should read

5.15 $$\delta \bar{S}+mN_{\text{tot}}\cdot E[\int_{0}^{T}\text{G}(\text{X},\text{V};f)\cdot \delta \text{X}\;\text{d}t+\sum_{\nu =1}^{M}\int_{0}^{T}{\text{g}}_{\nu }(\text{X},\text{V};f)\cdot \delta \text{X}\circ \text{d}W^{\nu }(t)]=0.$$

However, due to the presence of the expected value in this equation, part or all of the information about the Stratonovich integral term is lost, as we saw in (5.9) for instance. Therefore, if the domain (5.2) is chosen for $\bar{S}$, then the variational equations (5.13) or (5.15) do not determine a unique set of stochastic differential equations that need to be satisfied by the considered stochastic processes. Consequently, it is necessary to encode the missing information about the forcing terms ${\text{g}}_{\nu }$ in the definition of the action functional $\bar{S}$ by restricting its domain to a subset compatible with the considered collision operator. For the class of collision operators (5.3) a suitable choice of the domain is proposed in (5.5). For other collision operators appropriate domains will be nonlinear subspaces of (5.2), and they will be investigated in a follow-up work.

## 6. Variational principle for the Vlasov–Poisson equations

In the full Vlasov–Maxwell system, the scalar φ and vector **A** potentials are independent dynamic variables, and as such have to appear explicitly in the action functional alongside the stochastic processes **X**, **V** and **P**. In order to ensure the correct coupling between the stochastic processes and the electromagnetic field, an expected value was necessary in the definition of the action functional (5.1). This created a difficulty in deriving a variational principle, as pointed out in the remark following theorem 5.2. This difficulty can be circumvented for the Vlasov–Poisson equations because in this case the electrostatic potential φ is uniquely determined by the stochastic process **X**, as will be demonstrated below.

### (a) The collisional Vlasov–Poisson equations

The collisional Vlasov–Poisson equations

6.1 $$\frac{\partial f}{\partial t}+\text{v}\cdot \nabla_{x}f+\frac{q}{m}\text{E}\cdot \nabla_{v}f=C[f],$$

where

6.2*a* $$\text{E}=-\nabla_{x}\varphi$$

and

6.2*b* $$\Delta_{x}\varphi =-\rho ,$$

and the charge density *ρ* is given by (1.3), are an approximation of the Vlasov–Maxwell equations in the non-relativistic zero-magnetic field limit. The associated stochastic differential equations take the form

6.3*a* dX=V dt

and

6.3*b* $$\text{d}\text{V}=(\frac{q}{m}\text{E}(\text{X},t)+\text{G}(\text{X},\text{V};f))\;\text{d}t+\sum_{\nu =1}^{M}{\text{g}}_{\nu }(\text{X},\text{V};f)\circ \text{d}W^{\nu }(t).$$

The equations (2.4*b*), (6.2) and (6.3) form a stochastic reformulation of the Vlasov–Poisson equations. A stochastic particle discretization and the corresponding stochastic variational principle can be derived just like in §§3 and 4, respectively. Also, a variational principle analogous to the Lagrange–d’Alembert principle presented in §5 can be derived in a similar fashion. However, by doing so, one encounters the same difficulty with including the Stratonovich integral. In the case of the Vlasov–Poisson equations a different variational principle can be obtained by observing that the electrostatic potential φ can be expressed as a functional of the stochastic process **X**,

6.4 $$\varphi :R^{3}\times R\times C_{\Omega ,T}^{1}⟶R,$$

by solving Poisson’s equation (6.2*b*). Given the charge density function (2.4*b*) and specific boundary conditions, the solution of Poisson’s equation can be written using an appropriate Green’s function for the Laplacian. Assuming the spatial domain is unbounded, the standard Green’s function yields

6.5 $$\varphi (\text{x},t,\text{X})=\frac{1}{4\pi }\int_{R^{3}}\frac{\rho (\text{y},t)}{|\text{x}-\text{y}|}{\text{d}}^{3}\text{y}=\frac{qN_{\text{tot}}}{4\pi }E[\frac{1}{|\text{x}-\text{X}(t)|}].$$

From (6.2*a*) we have the electric field

6.6 $$\text{E}(\text{x},t,\text{X})=\frac{qN_{\text{tot}}}{4\pi }E[\frac{\text{x}-\text{X}(t)}{|\text{x}-\text{X}(t){|}^{3}}].$$

### (b) Action functional

Let us consider the action functional

6.7 $$\hat{S}:\Omega \times C_{\Omega ,T}^{1}\times C_{\Omega ,T}^{1}\times C_{\Omega ,T}^{0}\times C_{\Omega ,T}^{0}⟶R$$

defined by the formula

6.8 $$\hat{S}[\text{X},\text{Y},\text{V},\text{P}]=\int_{0}^{T}(\frac{m}{2}|\text{V}(t){|}^{2}-q\varphi (\text{X}(t),t,\text{Y})+\text{P}(t)\cdot (\dot{\text{X}}(t)-\text{V}(t)))\;\text{d}t,$$

where the electrostatic potential φ is given by (6.5). Note that similar to *S* in (4.4), the functional $\hat{S}$ is itself random, and can be viewed as the action functional of particles represented by the process **X** which are moving in the electric field generated by particles represented by the process Y. Similar to the calculations in §4b, the variations of $\hat{S}$ with respect to **X**, **V** and **P** are given by, respectively,

6.9*a* $$\begin{array}{rl} \delta_{\text{X}}\hat{S}[\text{X},\text{Y},\text{V},\text{P}] & =\text{P}(T)\cdot \delta \text{X}(T)-\text{P}(0)\cdot \delta \text{X}(0)\\ & \;-\int_{0}^{T}\delta \text{X}(t)\circ \text{d}\text{P}(t)+\int_{0}^{T}q\text{E}(\text{X}(t),t,\text{Y})\cdot \delta \text{X}(t)\;\text{d}t, \end{array}$$

6.9*b* $$\begin{array}{rl} \delta_{\text{V}}\hat{S}[\text{X},\text{Y},\text{V},\text{P}] & =\int_{0}^{T}(m\text{V}(t)-\text{P}(t))\cdot \delta \text{V}(t)\;\text{d}t \end{array}$$

6.9*c* $$\begin{array}{rl} \;\text{and}\;\delta_{\text{P}}\hat{S}[\text{X},\text{Y},\text{V},\text{P}] & =\int_{0}^{T}(\dot{\text{X}}(t)-\text{V}(t))\cdot \delta \text{P}(t)\;\text{d}t, \end{array}$$

where the electric field **E** is given by (6.6). Note that we are not considering variations with respect to Y. Let us for convenience define the joint variation of $\hat{S}$ with respect to **X**, **V** and **P** as

6.10 $$\delta_{\left(\text{X},\text{V},\text{P}\right)}\hat{S}=\delta_{\text{X}}\hat{S}+\delta_{\text{V}}\hat{S}+\delta_{\text{P}}\hat{S}.$$

### (c) The stochastic Lagrange–d’Alembert principle

In the following theorem, we formulate a variational principle for the system of equations (2.4*b*), (6.2) and (6.3). Note that E(X(t),t,X) is the electric field generated by a distribution of charged particles represented by the process **X** at time *t*, and evaluated at the random point x=X(t) in space. Furthermore, the notation $\delta_{\text{X}}\hat{S}[\text{X},\text{X},\text{V},\text{P}]$ means that the variation of $\hat{S}$ is evaluated for the arguments X,Y,V,P with Y=X.

**Theorem 6.1 (Stochastic Lagrange–d’Alembert principle for the VP equations).**

*Let* $X\in C_{\Omega ,T}^{1}$ *and* $V,P\in C_{\Omega ,T}^{0}$ *be stochastic processes, and let* φ(⋅,⋅,X)∈X(R) *be given by* (6.5). *Assume that* G(⋅,⋅;f) *and* $g_{\nu }(\cdot ,\cdot ;f)$ *for* ν=1,…,M *are* $C^{1}$ *functions of their arguments, where f is given by* (2.4*a*). *Then* X, V *and* P *satisfy the system of stochastic differential equations*

6.11*a* $$\begin{array}{rl} \dot{X}(t) & =V(t), \end{array}$$

6.11*b* $$\begin{array}{rl} P(t) & =mV(t) \end{array}$$

6.11*c* $$\begin{array}{rl} \;\text{and}\;\text{d}P(t) & =(qE(X(t),t,X)+m\;G(X(t),V(t);f))\;\text{d}t\\ & \;+m\sum_{\nu =1}^{M}g_{\nu }(X(t),V(t);f)\circ \text{d}W^{\nu }(t), \end{array}$$

*on the time interval* [0,T], *if and only if they satisfy the following variational principle*

6.12 $$\begin{array}{rl} & \delta_{\left(X,V,P\right)}\hat{S}[X,X,V,P]+m\int_{0}^{T}G(X,V;f)\cdot \delta X\;\text{d}t\\ & \;+m\sum_{\nu =1}^{M}\int_{0}^{T}g_{\nu }(X,V;f)\cdot \delta X\circ \text{d}W^{\nu }(t)=0, \end{array}$$

*for arbitrary variations* $\delta X\in C_{\Omega ,T}^{1}$, *and* $\delta V,\delta P\in C_{\Omega ,T}^{0}$, *with* δX(0)=δX(T)=0 *almost surely, where the action functional* $\hat{S}$ *is given by* (6.8).

**Proof.**

Analogous to the proof of theorem 4.2.

**Remark.**

It is straightforward to see that equations (6.11), together with (6.5) and (6.6), are equivalent to the system of equations (2.4*b*), (6.2) and (6.3). The Lagrange–d’Alembert principle (6.12) is unusual in that the variations of the action functional $\hat{S}$ with respect to the argument Y are omitted. Thanks to such a form, however, the action functional does not require an expected value, and the collisional effects can be correctly included. A similar idea to solve Poisson’s equation and plug the solution into the action functional was presented in [15], where the authors proposed a variational principle for the collisionless Vlasov–Poisson equations. In that approach the energy of the electric field was also included in the variational principle, and the variations were taken with respect to all arguments of the action functional. This approach could be adapted to the stochastic reformulation of the Vlasov–Poisson equations, but the corresponding action functional would have a form similar to (5.1), that is, it would need to contain an expected value, and therefore we would face a similar difficulty as for the Vlasov–Maxwell equations in §5b.

## 7. Summary and future work

In this work, we have considered novel stochastic formulations of the collisional Vlasov–Maxwell and Vlasov–Poisson equations, and we have identified new stochastic variational principles underlying these formulations. We have also proposed a stochastic particle method for the Vlasov–Maxwell equations and proved the corresponding stochastic variational principle.

Our work can be extended in several ways. The stochastic variational principle introduced in §4 can be used to construct stochastic variational PIC numerical algorithms for the collisional Vlasov–Maxwell and Vlasov–Poisson equations. Variational integrators are an important class of geometric integrators. This type of numerical scheme is based on discrete variational principles and provides a natural framework for the discretization of Lagrangian systems, including forced, dissipative or constrained ones. These methods have the advantage that they are symplectic when applied to systems without forcing, and in the presence of a symmetry, they satisfy a discrete version of Noether’s theorem. For this reason, they demonstrate superior performance in long-time simulations; see [3,75–84]. Variational integrators were introduced in the context of finite-dimensional mechanical systems, but were later generalized to Lagrangian field theories [2] and applied in many computations, for example in elasticity, electrodynamics, fluid dynamics, or plasma physics; see [10–12,65,72,85–87]. Stochastic variational integrators were first introduced in [35] and further studied in [18,34,37,39,40].

In §5, we have proposed a general action functional for the collisional Vlasov–Maxwell equations. However, we have also determined that in order to prove a relevant variational principle, the domain of this action functional has to be restricted in a way compatible with the collision operator of interest. We have shown that for a class of collision operators with constant diffusion terms, a suitable subdomain is an affine subspace (i.e. a submanifold). A natural continuation of our work would be to investigate submanifolds of (5.2) which are suitable for other collision operators.

Another aspect worth a more detailed investigation is the issue of existence and uniqueness of the solutions of the stochastic reformulations presented in this work, which are non-trivial systems of coupled stochastic and partial differential equations. This question is closely connected to the issue of existence and uniqueness of the solutions of the original collisional Vlasov–Maxwell and the Vlasov–Poisson equations. General results are available in the collisionless case (e.g. [88–90]), but the theory for the collisional equations is less developed (see [29,91–96] and references therein).

Furthermore, our stochastic Lagrange–d’Alembert approach could also be adapted to relativistic plasmas [97], and to variational principles with phase-space Lagrangians appearing in gyrokinetic [13,14,98] and guiding-centre theories [99–102]. In particular, considering stochastic extensions of the variational principles proposed in [99] could offer an alternative stochastic description of anomalous transport in magnetically confined plasmas [103,104].

Finally, as is typical for particle methods in general, the stochastic particle discretization proposed in §3 will require a large number of particles for accurate numerical simulations, which is computationally expensive. Structure-preserving model reduction methods [105,106] have been recently successfully applied to particle discretizations of the collisionless Vlasov equation [107]. It would be of great practical interest to combine our results with model reduction techniques in order to develop new efficient structure-preserving data-driven numerical methods for the collisional Vlasov–Maxwell equations.

## Data accessibility

This article has no additional data.

## Appendix A. The variations of the action functional S

We will define the variation of *S* with respect to the variation $\delta {\text{X}}_{a}\in C_{\Omega ,T}^{1}$ of the argument ${\text{X}}_{a}$ as

A 1 $$\delta_{{\text{X}}_{a}}S=\frac{\text{d}}{\text{d}\epsilon }{|}_{\epsilon =0}S[{\text{X}}_{1},\ldots ,{\text{X}}_{a}+\epsilon \delta {\text{X}}_{a},\ldots ,{\text{X}}_{N},{\text{V}}_{1},\ldots ,{\text{V}}_{N},{\text{P}}_{1},\ldots ,{\text{P}}_{N},\varphi ,\text{A}].$$

Since the potentials φ and **A** are $C^{2}$, and the processes ${\text{X}}_{b}$, ${\text{V}}_{b}$ and ${\text{P}}_{b}$ are almost surely continuous, we can use a dominated convergence argument to interchange the differentiation with respect to ϵ and integration with respect to *t* to obtain

A 2 $$\delta_{{\text{X}}_{a}}S=\frac{N_{\text{tot}}}{N}\int_{0}^{T}(-q\nabla_{x}\varphi ({\text{X}}_{a},t)\cdot \delta {\text{X}}_{a}+q\sum_{i,j=1}^{3}V^{j}\frac{\partial A^{j}}{\partial x^{i}}({\text{X}}_{a},t)\delta X_{a}^{i}+{\text{P}}_{a}\cdot \delta {\dot{\text{X}}}_{a})\;\text{d}t.$$

Since $\delta {\text{X}}_{a}$ is almost surely differentiable, we have that its stochastic differential is simply $\text{d}\delta {\text{X}}_{a}=\delta {\dot{\text{X}}}_{a}\;\text{d}t$. Furthermore, both $\delta {\text{X}}_{a}$ and ${\text{P}}_{a}$ are almost surely continuous semimartingales, therefore using the integration by parts formula for semimartingales [62] we can write

A 3 $$\int_{0}^{T}{\text{P}}_{a}\cdot \delta {\dot{\text{X}}}_{a}\;\text{d}t=\int_{0}^{T}{\text{P}}_{a}\circ \text{d}\delta {\text{X}}_{a}={\text{P}}_{a}(t)\cdot \delta {\text{X}}_{a}(t){|}_{0}^{T}-\int_{0}^{T}\delta {\text{X}}_{a}\circ \text{d}{\text{P}}_{a},$$

where the Stratonovich integrals are understood in the sense that $\int \delta {\text{X}}_{a}\circ \text{d}{\text{P}}_{a}=\sum_{i}\int \delta X_{a}^{i}\circ \text{d}P_{a}^{i}$. By substituting (A 3) in (A 2), we obtain (4.5*a*). Variations with respect to $\delta {\text{V}}_{a},\delta {\text{P}}_{a}\in C_{\Omega ,T}^{0}$ are defined analogously to (A 1). Similar computations (note that integration by parts is not necessary) yield (4.5*b*) and (4.5*c*), respectively.

The variation of *S* with respect to the variation $\delta \text{A}\in X_{0}(R^{3})$ of the vector potential **A** is defined as

A 4 $$\delta_{\text{A}}S=\frac{\text{d}}{\text{d}\epsilon }{|}_{\epsilon =0}S[{\text{X}}_{1},\ldots ,{\text{X}}_{N},{\text{V}}_{1},\ldots ,{\text{V}}_{N},{\text{P}}_{1},\ldots ,{\text{P}}_{N},\varphi ,\text{A}+\epsilon \delta \text{A}].$$

Switching the order of differentiation and integration, integrating by parts, and using the fact that δA is compactly supported, one arrives at (4.5*d*), where in the derivations we have used (3.2*c*) and

A 5 $$\begin{array}{rl} & \frac{N_{\text{tot}}}{N}\sum_{b=1}^{N}[\int_{0}^{T}q{\text{V}}_{b}(t)\cdot \delta \text{A}({\text{X}}_{b},t)\;\text{d}t]\\ & \;=\int_{0}^{T}\int_{R^{3}}\frac{qN_{\text{tot}}}{N}\sum_{b=1}^{N}[q{\text{V}}_{b}(t)\delta (\text{x}-{\text{X}}_{b}(t))]\cdot \delta \text{A}(\text{x},t)\;{\text{d}}^{3}\text{x}\text{d}t\\ & \;=\int_{0}^{T}\int_{R^{3}}\text{J}(\text{x},t)\cdot \delta \text{A}(\text{x},t)\;{\text{d}}^{3}\text{x}\text{d}t, \end{array}$$

and the remaining calculations are standard, and can be found in, e.g. [108,109]. The variation of *S* with respect to the variation $\delta \varphi \in X_{0}(R)$ of the scalar potential φ is defined in a similar fashion, and after similar calculations one obtains (4.5*e*).

## Appendix B. Proof of lemma 4.1

Proof. Suppose that (4.8) holds. Then (4.7) follows from the associativity property of the Stratonovich integral (see, e.g. the proof of theorem 2.1 in [37]). Conversely, assume that (4.7) is satisfied, and let us prove that (4.8) follows. Our reasoning very closely follows the proof of theorem 3.3 in [35]. Pick any time t∈[0,T]. We will use ${\text{e}}_{1}$, ${\text{e}}_{2}$ and ${\text{e}}_{3}$ to denote the standard Cartesian basis vectors for $R^{3}$. Pick a basis vector ${\text{e}}_{i}$. The condition (4.7) in particular holds for **Z**’s which are $C^{1}$ functions of time, i.e. non-random. The main idea of the proof is to construct a one-parameter family of $C^{1}$ functions ${\text{Z}}_{\epsilon }$ which converge to $1_{\left[0,t\right]}{\text{e}}_{i}$ as ϵ⟶0, and show that the integral in (4.7) converges almost surely to the integral in (4.8). Let us introduce the notation

B 1 $$\begin{array}{rl} I(\text{X},\text{V},\text{P},\text{Z}) & =\int_{0}^{T}(\text{Z}(\tau )\circ \text{d}\text{P}(\tau )-\text{R}(\text{X},\text{V})\cdot \text{Z}(\tau )\;\text{d}\tau \\ & -\sum_{\nu =1}^{M}{\text{r}}_{\nu }(\text{X},\text{V})\cdot \text{Z}(\tau )\circ \text{d}W^{\nu }(\tau )) \end{array}$$

and

B 2 $$\begin{array}{rl} I^{*}(\text{X},\text{V},\text{P}) & =\int_{0}^{T}(1_{\left[0,t\right]}{\text{e}}_{i}\circ \text{d}\text{P}(\tau )-\text{R}(\text{X},\text{V})\cdot 1_{\left[0,t\right]}{\text{e}}_{i}\;\text{d}\tau \\ & \;-\sum_{\nu =1}^{M}{\text{r}}_{\nu }(\text{X},\text{V})\cdot 1_{\left[0,t\right]}{\text{e}}_{i}\circ \text{d}W^{\nu }(\tau ))\\ & =\int_{0}^{t}(\text{d}P^{i}(\tau )-R^{i}(\text{X}(\tau ),\text{V}(\tau ))\;\text{d}\tau -\sum_{\nu =1}^{M}r_{\nu }^{i}(\text{X}(\tau ),\text{V}(\tau ))\circ \text{d}W^{\nu }(\tau )). \end{array}$$

Define the functions $h_{1}:[0,\epsilon ]⟶[0,1]$ and $h_{2}:[t-\epsilon ,t]⟶[0,1]$ by the formulae

B 3 $$h_{1}(\tau )=2\frac{\tau }{\epsilon }-\frac{\tau^{2}}{\epsilon^{2}}\;\text{and}\;h_{2}(\tau )=\left\{ \begin{array}{ll} -\frac{2}{\epsilon^{2}}{\left(\tau -t+\epsilon \right)}^{2}+1 & \text{if }t-\epsilon \leq \tau \leq t-\frac{\epsilon }{2},\\ \frac{2}{\epsilon^{2}}{\left(\tau -t+\epsilon \right)}^{2}-\frac{4}{\epsilon }(\tau -t+\epsilon )+2 & \text{if }t-\frac{\epsilon }{2}<\tau \leq t. \end{array}\right.$$

Note that $h_{1}(0)=h_{2}(t)=0$, $h_{1}(\epsilon )=h_{2}(t-\epsilon )=1$ and $h_{1}^{\prime }(\epsilon )=h_{2}^{\prime }(t-\epsilon )=h_{2}^{\prime }(t)=0$. Define further the family of functions ${\text{Z}}_{\epsilon }$ by the formula

B 4 $${\text{Z}}_{\epsilon }(\tau )=\left\{ \begin{array}{ll} h_{1}(\tau ){\text{e}}_{i} & \text{if }0\leq \tau \leq \epsilon ,\\ {\text{e}}_{i} & \text{if }\epsilon <\tau <t-\epsilon ,\\ h_{2}(\tau ){\text{e}}_{i} & \text{if }t-\epsilon \leq \tau \leq t,\\ 0 & \text{if }t<\tau \leq T. \end{array}\right.$$

It is easy to see that ${\text{Z}}_{\epsilon }$ is continuously differentiable^1^ on [0,T], and converges to $1_{\left[0,t\right]}{\text{e}}_{i}$ in the $L^{2}$ norm as ϵ goes to zero. Using (B 1)–(B 4), we have

B 5 $$\begin{array}{rl} & I^{*}(\text{X},\text{V},\text{P})-I(\text{X},\text{V},\text{P},{\text{Z}}_{\epsilon })\\ & \;=\int_{0}^{\epsilon }((1-h_{1}(\tau ))\circ \text{d}P^{i}(\tau )-(1-h_{1}(\tau ))R^{i}(\text{X},\text{V})\;\text{d}\tau -\sum_{\nu =1}^{M}(1-h_{1}(\tau ))r_{\nu }^{i}(\text{X},\text{V})\circ \text{d}W^{\nu })\\ & \;\;+\int_{t-\epsilon }^{t}((1-h_{2}(\tau ))\circ \text{d}P^{i}(\tau )-(1-h_{2}(\tau ))R^{i}(\text{X},\text{V})\;\text{d}\tau -\sum_{\nu =1}^{M}(1-h_{2}(\tau ))r_{\nu }^{i}(\text{X},\text{V})\circ \text{d}W^{\nu }). \end{array}$$

By definition, the Stratonovich integrals in (B 5) can be expressed in terms of the Itô integrals as

B 6 $$\begin{array}{rl} & \int_{0}^{\epsilon }(1-h_{1}(\tau ))r_{\nu }^{i}(\text{X},\text{V})\circ \text{d}W^{\nu }\\ & \;=\int_{0}^{\epsilon }(1-h_{1}(\tau ))r_{\nu }^{i}(\text{X},\text{V})\;\text{d}W^{\nu }+\frac{1}{2}[(1-h_{1}(\tau ))r_{\nu }^{i}(\text{X},\text{V}),W^{\nu }(\tau ){]}_{0}^{\epsilon }\\ \;\text{and}\; & \int_{t-\epsilon }^{t}(1-h_{2}(\tau ))r_{\nu }^{i}(\text{X},\text{V})\circ \text{d}W^{\nu }\\ & \;=\int_{t-\epsilon }^{t}(1-h_{2}(\tau ))r_{\nu }^{i}(\text{X},\text{V})\;\text{d}W^{\nu }+\frac{1}{2}[(1-h_{2}(\tau ))r_{\nu }^{i}(\text{X},\text{V}),W^{\nu }(\tau ){]}_{t-\epsilon }^{t}, \end{array}\}$$

for each ν=1,…,M, where [⋅,⋅] denotes the quadratic covariation process. Since the quadratic covariation of almost surely continuous semimartingales is itself a semimartingale with almost surely continuous paths (see theorem 23 in ch. II.6 of [62]), we have that

B 7 $$\begin{array}{rl} & [(1-h_{1}(\tau ))r_{\nu }^{i}(\text{X}(\tau ),\text{V}(\tau )),W^{\nu }(\tau ){]}_{0}^{\epsilon }\\ & \;⟶(1-h_{1}(0))r_{\nu }^{i}(\text{X}(0),\text{V}(0))W^{\nu }(0)=0\;\text{a.s. as }\epsilon ⟶0, \end{array}$$

since $W^{\nu }(0)=0$ almost surely. In a similar fashion, we show

B 8 $$[(1-h_{2}(\tau ))r_{\nu }^{i}(\text{X}(\tau ),\text{V}(\tau )),W^{\nu }(\tau ){]}_{t-\epsilon }^{t}⟶0\;\text{a.s. as }\epsilon ⟶0.$$

Using (B 5) and (B 6), we have the estimate

B 9 $$\begin{array}{rl} & |I^{*}(\text{X},\text{V},\text{P})-I(\text{X},\text{V},\text{P},{\text{Z}}_{\epsilon })|\\ & \;\leq \underset{\Gamma_{1}}{\underbrace{|\int_{0}^{\epsilon }((1-h_{1}(\tau ))\circ \text{d}P^{i}(\tau )-(1-h_{1}(\tau ))R^{i}(\text{X},\text{V})\;\text{d}\tau -\sum_{\nu =1}^{M}(1-h_{1}(\tau ))r_{\nu }^{i}(\text{X},\text{V})\;\text{d}W^{\nu })|}}\\ & \;+\underset{\Gamma_{2}}{\underbrace{|\int_{t-\epsilon }^{t}((1-h_{2}(\tau ))\circ \text{d}P^{i}(\tau )-(1-h_{2}(\tau ))R^{i}(\text{X},\text{V})\;\text{d}\tau -\sum_{\nu =1}^{M}(1-h_{2}(\tau ))r_{\nu }^{i}(\text{X},\text{V})\;\text{d}W^{\nu })|}}\\ & \;+\frac{1}{2}\sum_{\nu =1}^{M}|[(1-h_{1}(\tau ))r_{\nu }^{i}(\text{X},\text{V}),W^{\nu }(\tau ){]}_{0}^{\epsilon }|+\frac{1}{2}\sum_{\nu =1}^{M}|[(1-h_{2}(\tau ))r_{\nu }^{i}(\text{X},\text{V}),W^{\nu }(\tau ){]}_{t-\epsilon }^{t}|. \end{array}$$

By bounding the integrands and using the Itô isometry theorem, it is shown in [35] that $\Gamma_{1}⟶0$ and $\Gamma_{2}⟶0$ in mean-square as ϵ⟶0, and consequently, by invoking the Borel–Cantelli lemma, there exists a subsequence $\left(\epsilon_{n}\right)$ such that $\epsilon_{n}⟶0$ as n⟶∞, for which $\Gamma_{1}⟶0$ and $\Gamma_{2}⟶0$ almost surely. Together with (B 7) and (B 8), this means that $I(\text{X},\text{V},\text{P},{\text{Z}}_{\epsilon_{n}})⟶I^{*}(\text{X},\text{V},\text{P})$ almost surely. Given the assumption (4.7), we have that $I^{*}(\text{X},\text{V},\text{P})=0$ almost surely, which completes the proof.
